# Polygenic risk score and 20-year prostate cancer-specific mortality and survival

**DOI:** 10.1038/s43856-026-01603-9

**Published:** 2026-04-24

**Authors:** Anna Plym, Anqi Wang, Konrad H. Stopsack, Yiwen Zhang, Aris Baras, Regeneron Genetics Center, Aris Baras, Isabel Drake, Mark Clements, Kathryn L. Penney, Edward Giovannucci, Adam S. Kibel, Lorelei A. Mucci, Fredrik Wiklund

**Affiliations:** 1 Department of Medical Epidemiology and Biostatistics, Karolinska Institutet, Stockholm, Sweden; 2 Department of Epidemiology, Harvard T. H. Chan School of Public Health, Boston, MA USA; 3 Department of Urology, Mass General Brigham, Harvard Medical School, Boston, MA USA; 4 Department of Epidemiologic Methods and Etiologic Research, Leibniz Institute for Prevention Research and Epidemiology – BIPS and Faculty of Human and Health Sciences, University of Bremen, Bremen, Germany; 5 Regeneron Genetics Center, Tarrytown, New York, USA; 6 Department of Clinical Sciences in Malmö, Lund University, Malmö, Sweden; 7 Skåne University Hospital, Malmö, Sweden; 8 Channing Division of Network Medicine, Department of Medicine, Brigham and Women’s Hospital and Harvard Medical School, Boston, MA USA; 9 Department of Nutrition, Harvard T.H. Chan School of Public Health, Boston, MA USA

**Keywords:** Prostate, Prognostic markers, Genetic variation

## Abstract

**Background:**

Polygenic risk scores (PRSs) strongly discriminate for prostate cancer risk at the population level. The role of these genetic scores in determining prostate cancer survival is unclear, potentially due to methodological issues.

**Methods:**

We included 19,607 men from the Malmö Diet and Cancer Study (MDCS) and the Health Professionals Follow-up Study (HPFS) and analyzed 20-year incidence and mortality (from full cohort analyses) and survival (from case-only analyses) according to a 451-variant PRS. For most of the men included, early access to prostate-specific antigen (PSA) testing was limited.

**Results:**

In full cohort analyses, a PRS at or above the median (vs. below the median) shows a strong association with prostate cancer incidence (hazard ratio (HR) 3.02, 95% CI 2.78-3.28) and, somewhat stronger, with prostate cancer mortality (HR 3.26, 95% CI 2.63-4.04). As expected, case-only survival analyses of prostate cancer death show a similar direction (HR 1.21, 95% CI 0.98-1.50), which becomes stronger when excluding variants linked to PSA (HR 1.25, 95% CI, 1.01-1.54), in particular in the age group 65-74 years at diagnosis (HR 1.72, 95% CI 1.21-2.45). In the other age groups, HRs are close to or below 1. This indicates that standard case-only survival estimates may not accurately reflect risk across age, consistent with age-dependent selection mechanisms, and further points to an influence from disease detection.

**Conclusions:**

Overall, these findings support that inherited genetic risk captured by the 451-variant PRS may have an influence on prostate cancer survival.

## Plain Language Summary

Inherited genetic factors, in particular when combined into a polygenic risk score, can be used to determine risk for prostate cancer. However, in men already diagnosed with prostate cancer, the role of such scores is unclear. In this study, we examined if a previously developed polygenic risk score for prostate cancer risk can be informative for survival. We found that for some age groups in the two included cohorts of nearly 20,000 men, a high genetic risk score was associated with worse prostate cancer survival. We also identified individual genetic factors that could play a role, although the relevance for men diagnosed today will need more research. Our study further highlights methodological concerns in studies of inherited genetic risk and cancer survival.

## Abstract (web summary)

Plym et al. investigate the role of a 451‑variant polygenic risk score in determining prostate cancer survival. The findings indicate a link between a higher polygenic risk score and worse survival, but also reveal that survival analyses of inherited genetic risk variants are sensitive to bias and influences from age and case selection.

## Introduction

Prostate cancer risk is strongly influenced by genetic factors and has an estimated heritability of 58%^1^. In addition to family history which is an established risk factor^2^, genome-wide association studies (GWASs) in multiethnic populations have identified 451 inherited genetic risk variants for prostate cancer susceptibility^3^. Combined into a polygenic risk score (PRS), these genetic risk variants show strong and robust associations with prostate cancer incidence across diverse ancestry groups. The PRS is also strongly associated with prostate cancer mortality at the population level^4,5^ – however, such an association could be driven by the higher incidence even if the PRS has no direct prognostic influence on prostate cancer-specific death^6^.

In contrast to the advancements in identifying inherited genetic risk variants for prostate cancer susceptibility, less progress has been made when it comes to understanding the role of inherited factors in prostate cancer-specific survival. Rare alterations within *BRCA2* and other DNA repair genes have been recognized to be of importance in some clinical settings^7^, but aside from that there is no consensus. Previous GWASs of prostate cancer-specific survival have identified potential risk variants^8,9^ but findings have generally been hard to replicate. PRSs for prostate cancer susceptibility have not shown any clear link to prostate cancer aggressiveness or survival^10,11^ although a 400-variant of the latest 451-variant PRS – which excludes variants linked to prostate-specific antigen (PSA) levels – shows marginally stronger but consistent and statistically significant associations for aggressive vs. non-aggressive disease across several populations (odds ratio of 1.04 to 1.10 per standard deviation increase of the PRS)^3^. There are known methodological concerns with case-only survival analyses such as collider stratification bias^12,13^ and lead-time bias which could have contributed to why studies have failed to validate genetic factors relevant for prostate cancer-specific survival.

Because of the interest in improving post-diagnostic risk stratification through incorporation of genetic factors and since potential methodological concerns have previously not received much attention within this field, we report on the role of PRS on prostate cancer mortality (full cohort data) and survival (case-only data) from two cohorts of men. Based on 20 years of follow-up for prostate cancer death, our analyses show that while case-only analyses may indicate the direction – here suggesting a higher risk of death among men with higher PRS – they are vulnerable to several influences related to detection, treatment, and age. Overall, such studies require more careful interpretation than previously recognized.

## Methods

### Study populations

We included men with existing genotype data from the Malmö Diet and Cancer Study (MDCS) and the Health Professionals Follow-up Study (HPFS), which are similar in design and follow-up, and for a subset of the analyses involving individual genetic variants to strengthen these analyses, men from the Physicians’ Health Study (PHS). The MDCS is a population-based prospective cohort study including 12,120 men recruited and with blood samples collected between 1991 to 1996 at ages 46 to 73 years^14^. The current analysis was restricted to those with genetic data without a prostate cancer diagnosis at cohort baseline and who had complete data (10,270 men). At study entry and for parts of the follow-up, PSA testing was not widely available and the MDCS is considered to be a mostly unscreened cohort^15^.

The HPFS is a prospective cohort study of 51,529 U.S. male health professionals, recruited in 1986 at ages 40 to 75 years^16^. Through previously performed nested case-control studies, genotype data are available for 10,917 of these men from blood samples collected 1993-1999 and buccal cell samples 2005-2006, with the vast majority (99%) self-reporting as White^17^. The current analysis was restricted to genotyped men with complete data who were alive and without a prostate cancer diagnosis in 1996, when detailed information on family history of cancer was collected (9337 men). PSA testing was not available at cohort baseline (1986), but the uptake of PSA testing among men in the HPFS is high (>80% at entry into the analysis)^5^. From the PHS, which began in 1982 and recruited U.S. male physicians, we include 1968 genotyped men of whom 853 were prostate cancer cases through January 2010^18^.

### Genetic risk

As described previously^19^ PRSs were calculated for all men as the sum of risk allele dosages weighted by the log odds ratios from the most recent underlying multi-ancestry GWAS^3^. We focused on the full 451-variant PRS (of which 434 variants were available in all cohorts and included for analysis), the 400-variant PRS excluding variants related to levels of PSA, and for additional investigation, a PRS only including a subset of the 434 variants with the strongest association with prostate cancer mortality based on the log(hazard ratio) and p-values (<0.01) from meta-analyses of all three cohorts (MDCS, HPFS, and PHS) (Supplementary Data 1). Based on our previous work^5^, we categorized men in the MDCS and the HPFS according to the distribution of the PRS in the full cohorts (PRS 0-50% and 50-100%, and in additional examinations, according to PRS quintiles). We further combined the PRS with first-degree family history of prostate cancer (HPFS) or cancer in the father (MDCS).

### Lifestyle and clinical factors

Covariates included smoking, body mass index (BMI) and clinical factors. Men were categorized according to being a current or past smoker quitting <10 years ago (yes/no) and having a BMI ≥ 30 kg/m^2^ (yes/no), reported at the start of follow-up. Clinical factors included age and calendar year at diagnosis; modified National Comprehensive Cancer Network (NCCN) prostate cancer risk groups; and primary treatment strategy. The risk groups were low risk (T1–T2/T2a, Gleason score ≤6, and PSA < 10 ng/ml), intermediate risk (T2b–T2c or Gleason score 7 or PSA 10– < 20 ng/ml), high risk including locally advanced and regionally metastatic prostate cancer (T3-T4 or Gleason score 8–10 or PSA ≥ 20 ng/ml), and metastatic disease (M1). Because detailed treatment data were only partly available, men were grouped into conservative (active surveillance or watchful waiting), curative-intent (radical prostatectomy or radiotherapy), and hormonal therapy or non-curative management.

### Cancer outcomes

Two outcomes were studied: a diagnosis of prostate cancer and prostate cancer death. For the MDCS, outcome data were available through a record linkage with the National Prostate Cancer Register and the Swedish Cause of Death Register. For the HPFS and the PHS, outcome data were available through follow-up questionnaires and subsequently confirmed through medical record reviews, and by searches in the National Death Index and reports of next of kin (causes of death were assigned by a physician committee blinded to any exposure data). For all three cohorts, outcome data are near complete (>96%).

### Statistical analysis

Both full cohort incidence and mortality analyses, and case-only survival analyses were conducted. In the full cohort analyses, start of follow-up was defined as the age of study entry in the MDCS (1991 to 1996) or the age at the completion of the 1996-questionnaire (or age at DNA collection, if later) in the HPFS. In the case-only analyses, the start of follow-up was the date of cancer diagnosis (mimicking the standard approach for such analyses). Men were followed until prostate cancer diagnosis (for incidence analyses) or prostate cancer-specific death (for mortality/survival analyses), death from other causes, emigration, or end of follow-up. For the HPFS, the end of follow-up was January 2017 (incidence) and January 1^st^ 2019 (mortality). For the MDCS, the end of follow-up was December 31^st^ 2019. For PHS, the end of follow-up was January 2010.

Cox proportional hazard regression was used to estimate hazard ratios (HRs) and 95% confidence intervals (CIs) for the association between the risk factors and the outcomes. For men in the HPFS and the PHS, the full cohort analysis was weighted to account for the underlying selection of men for genotyping, as previously described^18^. In the case-only analysis, we fitted both age-adjusted (continuous age at diagnosis variable) and age-stratified models (using interaction terms with three categories for age at diagnosis: <65, 65-74, and ≥75 years) where the upper limit was defined based on our previous work^5^. In separate models, we included adjustment for the lifestyle and clinical factors. Adjustment for genetic ancestry was considered but not included in the final model given nearly identical estimates. Estimates from the cohorts were combined using random effects meta-analysis.

For cases, we further calculated the 20-year absolute risk of prostate cancer death using the non-parametric (unadjusted) Aalen-Johansen estimator of the cumulative incidence function^20^, treating death due to other causes as a competing event and using four age categories instead of three.

All analyses were performed in R versions 4.2.3 and 4.4.0.

All participants in each study gave written informed consent, and the protocols for the included cohort studies and secondary analyses were approved by the regional ethical review board in Lund, Sweden and the institutional review boards of Brigham and Women’s Hospital and Harvard T.H. Chan School of Public Health, and those of participating registries as required.

### Reporting summary

Further information on research design is available in the Nature Portfolio Reporting Summary linked to this article.

## Results

The 10,270 men in the MDCS and the 9337 men in the HPFS had a median (IQR) age of 59.0 (53.0 to 64.7) and 65.1 (58.0 to 71.8) years at start of follow-up, and were followed for a median (IQR) time until prostate cancer death of 24.0 (16.0 to 25.9) and 23.4 (13.9 to 25.0) years, respectively. During follow-up, 1574 (MDCS) and 1777 (HPFS) men were diagnosed, and 288 (MDCS) and 156 (HPFS) men died of prostate cancer. Among the diagnosed cases, men in the PRS 50-100% category were over-represented, and further diagnosed at a younger age compared with men in the PRS 0-50% category (Table 1).

**Table 1:** Characteristics of prostate cancer cases in the Malmö Diet and Cancer Study (MDCS, *n* = 1574) and the Health Professionals Follow-up Study (HPFS, *n* = 1777) according to the 451-variant PRS

	MDCS		HPFS	
	PRS 0-50% (*n* = 431)	PRS 50-100% (*n* = 1143)	PRS 0-50% (*n* = 483)	PRS 50-100% (*n* = 1294)
Age at diagnosis, year, median [IQR]	72.8 [67.9, 77.2]	71.7 [67.0, 76.1]	73.1 [67.1, 77.6]	70.5 [65.4, 75.7]
Age at diagnosis, year, n (%)				
50-64	50 (11.6)	197 (17.2)	86 (17.8)	290 (22.4)
65-74	215 (49.9)	588 (51.4)	203 (42.0)	639 (49.4)
≥ 75	166 (38.5)	358 (31.3)	194 (40.2)	365 (28.2)
Age at prostate cancer death, year, median [IQR]	80.7 [75.0, 86.7]	80.0 [74.8, 85.5]	83.8 [76.7, 89.0]	82.7 [76.4, 87.4]
Age at end follow-up, year, median [IQR]	82.1 [77.2, 86.6]	80.9 [76.5, 85.3]	84.8 [78.8, 89.1]	83.3 [77.9, 88.1]
Year of diagnosis, n (%)				
<2000	76 (17.6)	201 (17.6)	114 (23.6)	342 (26.4)
2000-2004	115 (26.7)	353 (30.9)	167 (34.6)	466 (36.0)
2005-2009	84 (19.5)	247 (21.6)	134 (27.7)	366 (28.3)
≥ 2010	156 (36.2)	342 (29.9)	68 (14.1)	120 (9.3)
NCCN risk groups, n (%)				
Low	118 (31.0)	275 (26.6)	172 (38.1)	440 (35.7)
Intermediate	88 (23.1)	289 (28.0)	189 (41.9)	557 (45.2)
High	140 (36.7)	339 (32.8)	81 (18.0)	214 (17.4)
Distant metastasis	35 (9.2)	130 (12.6)	9 (2.0)	21 (1.7)
Missing	50	110	32	62
Primary treatment strategy, n (%)				
Conservative	109 (27.9)	264 (25.4)	57 (12.6)	114 (9.4)
Curative	159 (40.8)	450 (43.3)	353 (78.1)	1004 (82.6)
Hormone therapy/other	122 (31.3)	326 (31.3)	42 (9.3)	98 (8.1)
Missing	41	103	31	78
Family history ^a^	95 (22.0)	286 (25.0)	85 (17.6)	273 (21.1)
Current smoker or quit <10 years	149 (34.6)	398 (34.8)	43 (8.9)	127 (9.8)
BMI ≥ 30 kg/m^2^	50 (11.6)	127 (11.1)	46 (9.5)	140 (10.8)

*BMI* body mass index, *HPFS* Health Professionals Follow-up Study, *IQR* interquartile range, *NCCN* National Comprehensive Cancer Network, *MDCS* Malmö Diet and Cancer Study, *PRS* polygenic risk score. a. Family history indicates a first-degree family history of prostate cancer (HPFS) or any history of cancer in the father (MDCS).

In full cohort analyses, having a 451-variant PRS at or above the median (vs. below the median) was strongly associated with prostate cancer incidence (HR 3.02, 95% CI 2.78–3.28), and as expected but somewhat stronger, with prostate cancer mortality (HR 3.26, 95% CI 2.63–4.04) (Table 2). In the case-only survival analysis, the age-adjusted HRs for the same comparison was 1.21 (95% CI 0.98–1.50). For the 400-variant PRS, the HRs for incidence and mortality were somewhat weaker than for the 451-variant PRS, whereas the HR in the case-only survival analysis showed a somewhat stronger association (HR 1.25, 95% CI, 1.01–1.54). A more detailed PRS categorization (in quintiles, comparing PRS 80-100% vs. PRS 40–60%) showed a stronger association for the 400-variant PRS in the case-only survival analysis (HR 1.35, 95% CI 1.01–1.79) (Supplementary Table 1). The estimates were similar for both cohorts (Supplementary Table 2).

**Table 2:** Different versions of the 451-variant PRS in relation to prostate cancer diagnosis (incidence) and prostate cancer death (mortality/survival), from meta-analyses of the MDCS (*n* = 10,270) and the HPFS (*n* = 9337) (see Supplementary Table 2 for cohort-specific estimates)

	Incidence (full cohort)		Mortality (full cohort)		Survival (case-only)	
	Events/pyrs	HR (95% CI) ^a^	Events/pyrs	HR (95% CI) ^a^	Events/pyrs	HR (95% CI) ^b^
451-variants PRS						
0-50%	914/291449	1 (Ref.)	113/316640	1 (Ref.)	113/9435	1 (Ref.)
50-100%	2437/253874	3.02 (2.78-3.28)	331/293246	3.26 (2.63-4.04)	331/26435	1.21 (0.98-1.50)
400-variant PRS (excluding PSA variants)						
0-50%	1007/287705	1 (Ref.)	122/313414	1 (Ref.)	122/10637	1 (Ref.)
50-100%	2344/257619	2.61 (2.39-2.84)	322/296471	2.87 (2.32-3.54)	322/25233	1.25 (1.01-1.54)
16-variant PRS for mortality						
0-50%	1397/279449	1 (Ref.)	147/309351	1 (Ref.)	147/15063	1 (Ref.)
50-100%	1954/265876	1.47 (1.29-1.68)	297/300535	2.08 (1.60-2.70)	297/20808	1.47 (1.20-1.81)

*CI* confidence interval, *HPFS* Health Professionals Follow-up Study, *HR* hazard ratio, *MDCS* Malmö Diet and Cancer Study, *PRS* polygenic risk score, pyrs person-years, *Ref.* reference. a. From models using age as the underlying timescale. b. From models using time since diagnosis as the underlying timescale, with additional adjustment for age at diagnosis.

A similar pattern of a stronger association with survival when the incidence association is weaker was observed for the third PRS examined (Table 2). In this PRS, we filtered for variants with strong associations with mortality in full cohort analyses (p-value < 0.01), leaving 16 variants as further discussed below. Whereas the case-only HR was stronger for this PRS than for the other PRSs (HR 1.47, 95% CI, 1.20–1.81), it should be noted that this estimate may be inflated because it was generated, at least in parts, from the same data sets. Crosswise comparisons indicated some replicability of the approach, although standard case-only analyses may not provide the full picture for replicability (Supplementary Table 3).

**Fig. 1:**
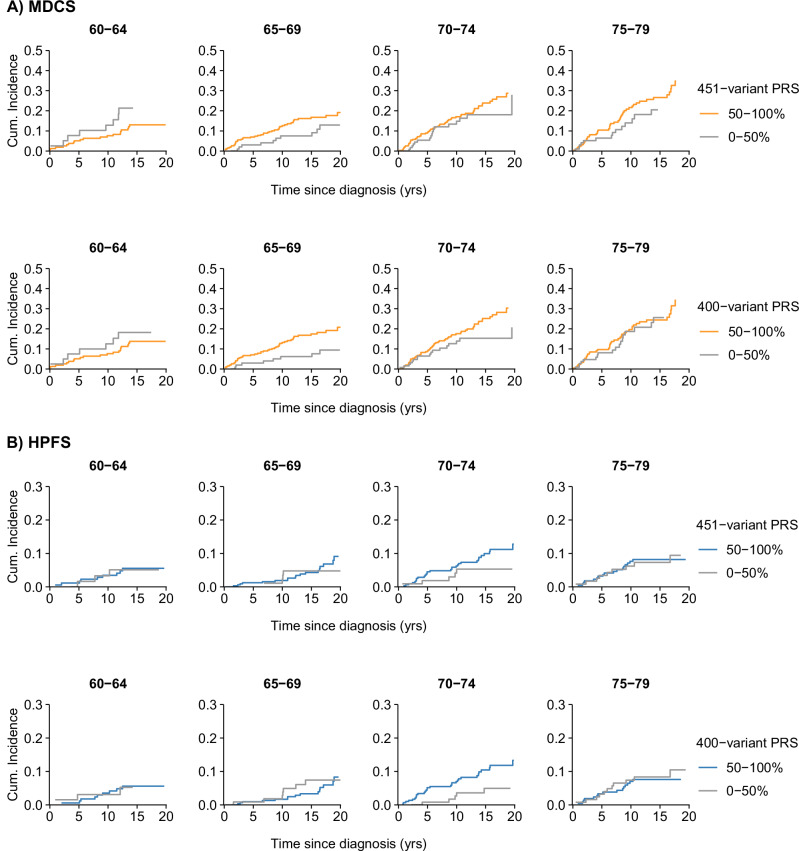
20-year absolute risk (cumulative incidence) of prostate cancer death for the 451-variant and the 400-variant PRS, stratified by age at diagnosis among prostate cancer cases. **A** Cases from the Malmö Diet and Cancer Study (MDCS, *n* = 1574). **B** Cases from the Health Professionals Follow-up Study (HPFS, *n* = 1777).

For all PRS versions, age at diagnosis-stratified case-only survival analyses showed considerably stronger positive associations for the middle age group, to which 49% of all cases belonged to, potentially indicating that the overall case-only survival estimate is biased or does not accurately reflect the risk across different ages (Table 3). For example, for the 400-variant PRS, the HR was 1.72 (95% CI 1.21-2.45) among men diagnosed between ages 65 to 74 years. In contrast, the HRs for the younger and older age group were around or below 1: 0.70 (95% CI 0.40-1.23) for the younger and 1.05 (95% CI 0.78-1.41) for the older age group. Cohort-specific estimates were similar (Supplementary Table 4), with p-values for interaction of 0.01 (MDCS) and 0.29 (HPFS), respectively. The age-specific patterns and strength of association remained similar, or became stronger, after including family history and with additional adjustment for smoking, BMI, and clinical factors and treatment (Supplementary Tables 5-7). In the examination of 20-year absolute risks, the same pattern was observed (Fig. 1). Among men in the MDCS, a higher risk of prostate cancer death with a PRS at or above the median was most notable in the ages 65-69 and 70-74 years, and for the HPFS, in the age group 70-74 years.

**Table 3:** Different versions of the 451-variant PRS in relation to prostate cancer death in case-only survival analyses stratified by age at diagnosis, from meta-analyses of the MDCS (*n* = 1574) and the HPFS (*n* = 1777) (see Supplementary Table 4 and Fig. 1 for cohort-specific estimates)

	Survival (case-only)					
	Age <65 years		Age 65–74 years		Age ≥ 75 years	
	Events/pyrs	HR (95% CI) ^a^	Events/pyrs	HR (95% CI) ^a^	Events/pyrs	HR (95% CI) ^a^
451-variants PRS						
0-50%	17/1887	1 (Ref.)	38/4842	1 (Ref.)	58/2706	1 (Ref.)
50-100%	42/7286	0.63 (0.36-1.11)	154/13915	1.50 (1.05-2.14)	135/5234	1.13 (0.83-1.54)
400-variant PRS (excluding PSA variants)						
0-50%	17/2125	1 (Ref.)	39/5559	1 (Ref.)	66/2953	1 (Ref.)
50-100%	42/7047	0.70 (0.40-1.23)	153/13200	1.72 (1.21-2.45)	127/4987	1.05 (0.78-1.41)
16-variant PRS for mortality						
0-50%	24/3585	1 (Ref.)	56/8004	1 (Ref.)	67/3474	1 (Ref.)
50-100%	35/5587	0.90 (0.53-1.51)	136/10753	1.72 (1.26-2.34)	126/4466	1.40 (0.92-2.13)

*CI* confidence interval, *HPFS* Health Professionals Follow-up Study, *HR* hazard ratio, *MDCS* Malmö Diet and Cancer Study, *PRS* polygenic risk score, pyrs person-years, *Ref.* reference. a. From models stratified by age at diagnosis.

**Fig. 2:**
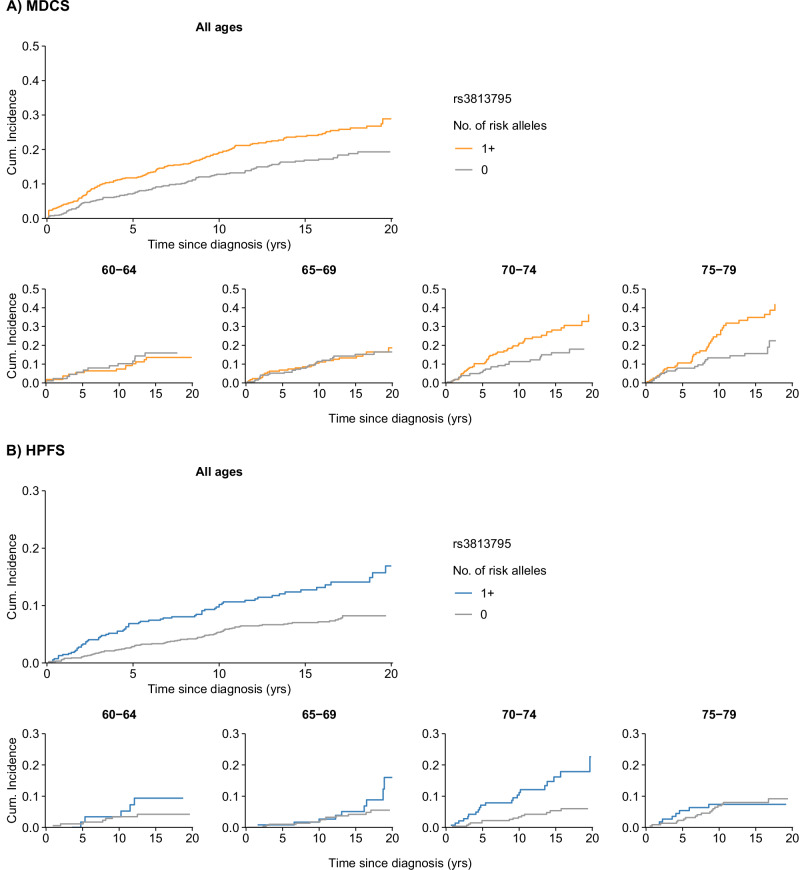
20-year absolute risk (cumulative incidence) of prostate cancer death for number of risk alleles for rs3813795 within *SYTL1*, overall and stratified by age at diagnosis among prostate cancer cases. **A** Cases from the Malmö Diet and Cancer Study (MDCS, *n* = 1574). **B** Cases from the Health Professionals Follow-up Study (HPFS, *n* = 1777).

Among the 16 variants with the strongest association with prostate cancer mortality, we observed three variants in the 8q24 region, two variants in the 4q24 region (mapped to *TET2*), and three variants altering protein structure within *SYTL1*, *ANO7*, and *CHEK2* (Table 4). Similar to above observations, variants with a less strong association with incidence showed a tendency to also show a stronger association in case-only analyses, with the variant within *SYTL1* showing the strongest association (p-value = 0.0003) (Fig. 2), followed by variants in *RNLS, ITPK, CDKN2B-AS1*, and *GRHL1*.

**Table 4:** Risk variants (*N* = 16) within the PRS with the strongest association with prostate cancer mortality (*P*-value < 0.01) in meta-analyses of data from all three cohorts (MDCS, *n* = 10,270, HPFS, *n* = 9337, and PHS, *n* = 1986), and their association with incidence, mortality, and survival (HR per risk allele increase)

rsID	Chromosome and position (GRCh37)	RAF ^a^	Nearest gene ^a^	Incidence (full cohort)		Mortality (full cohort)		Survival (case-only)	
				HR (95% CI) ^b^	*p*-value	HR (95% CI) ^b^	*p*-value	HR (95% CI) ^c^	*p*-value
rs3813795	1:27679797	0.308	*SYTL1*	1.02 (0.96-1.08)	4.8×10^-1^	1.26 (1.11-1.42)	2.6×10^-4^	1.26 (1.11-1.42)	3.0×10^-4^
rs141153466	1:93312203	0.0003	*DIPK1A*	7.57 (0.47-122.46)	1.5×10^-1^	14.08 (3.72-53.28)	9.8×10^-5^	– ^d^	– ^d^
rs73913932	2:10094526	0.072	*GRHL1*	1.06 (0.95-1.17)	3.0×10^-1^	1.42 (1.17-1.74)	5.3×10^-4^	1.24 (1.02-1.51)	3.2×10^-2^
rs76832527	2:242157241	0.173	*ANO7*	1.17 (1.09-1.26)	3.7×10^-5^	1.27 (1.08-1.51)	5.0×10^-3^	1.04 (0.81-1.32)	7.7×10^-1^
rs4527381	3:87221370	0.504	*LINC00506;MIR4795*	1.13 (1.08-1.20)	2.6×10^-6^	1.20 (1.07-1.35)	2.0×10^-3^	1.07 (0.96-1.20)	2.3×10^-1^
rs10007915	4:106065308	0.591	*CXXC4-AS1;TET2*	1.13 (1.07-1.19)	5.9×10^-6^	1.27 (1.13-1.44)	9.8×10^-5^	1.13 (1.00-1.26)	4.4×10^-2^
rs34316731	4:106078344	0.867	*TET2*	1.19 (1.09-1.29)	7.0×10^-5^	1.32 (1.09-1.60)	4.2×10^-3^	1.12 (0.93-1.35)	2.5×10^-1^
rs77541621	8:128077146	0.024	*PCAT1;PCAT2*	1.86 (1.60-2.17)	1.6×10^-15^	1.81 (1.30-2.52)	4.2×10^-4^	0.96 (0.70-1.30)	7.8×10^-1^
rs18337302	8:128104117	0.007	*PRNCR1*	2.49 (1.96-3.16)	9.5×10^-14^	3.64 (1.92-6.92)	7.7×10^-5^	1.54 (0.80-2.97)	1.9×10^-1^
rs11986220	8:128531689	0.110	*CASC8;CASC11*	1.42 (1.00-2.02)	5.0×10^-2^	1.47 (1.17-1.84)	7.6×10^-4^	1.06 (0.90-1.26)	4.9×10^-1^
rs17694493	9:22041998	0.136	*CDKN2B-AS1*	1.05 (0.97-1.13)	2.3×10^-1^	1.29 (1.11-1.51)	1.3×10^-3^	1.19 (1.02-1.39)	2.5×10^-2^
rs792207	10:90167065	0.413	*RNLS*	1.06 (1.01-1.12)	2.9×10^-2^	1.21 (1.08-1.36)	1.0×10^-3^	1.15 (1.03-1.29)	1.5×10^-2^
rs11228580	11:69002342	0.161	*LOC338694;MYEOV*	1.20 (1.06-1.36)	4.0×10^-3^	1.22 (1.06-1.42)	7.7×10^-3^	1.07 (0.92-1.23)	3.8×10^-1^
rs12586382	14:93464018	0.123	*ITPK1*	1.07 (0.99-1.16)	9.7×10^-2^	1.30 (1.10-1.53)	1.7×10^-3^	1.27 (1.03-1.56)	2.4×10^-2^
rs760118	16:4940994	0.563	*PPL*	1.07 (1.01-1.13)	1.8×10^-2^	1.17 (1.04-1.32)	9.9×10^-3^	1.09 (0.97-1.23)	1.6×10^-1^
rs55560770	22:29091856	0.003	*CHEK2*	1.89 (1.34-2.66)	2.8×10^-4^	2.51 (1.26-5.04)	9.3×10^-3^	1.37 (0.71-2.63)	3.5×10^-1^

*CI* confidence interval, *HR* hazard ratio, *PRS* polygenic risk score, *RAF* risk allele frequency, *Ref.* reference. a. RAF (European ancestry) and nearest gene from Wang et al., Nature Genetics, 2023. b. From models using age as the underlying timescale. c. From models using time since diagnosis as the underlying timescale, with additional adjustment for age at diagnosis. d. No meaningful estimation due to low RAF.

Estimates for all variants in the PRS can be found in Supplementary Data 1. *P*-values are two-sided from Wald tests, without adjustment for multiple comparisons.

## Discussion

With an expected increase in the use of PRS for risk stratification of prostate cancer and other diseases, understanding if having a high PRS has any influence on prognosis after diagnosis will become increasingly relevant for both patients and treating physicians^11,21,22^. Until now, the scope of potential problems or biases that may arise when attempting to answer such questions have not been fully understood nor picked up by the wider medical community. The present study shows that while the standard case-only survival analysis of the PRS was only indicative of an increased post-diagnostic risk of dying from prostate cancer (HRs of 1.21 to 1.25, depending on the version), stratification by age at diagnosis showed stronger associations, indicating up to 2-fold higher risks among patients in the large age group 65 to 74 years, also after controlling for clinical factors and treatment. Such an interaction is potentially explained by the strong relationship of the PRS with disease incidence and age-related mechanisms, as further discussed below. Overall, our results point to that the PRS, or variants within the PRS, could have a link to worse prostate cancer outcomes, at least in men not screened with PSA from an early age which was the case for men in both the MDCS and the HPFS.

Several mechanisms may provide an explanation for our age-at-diagnosis-diverging results presented in Table 3 and Fig. 1. Early detection and treatment, which would presumably attenuate the consequences of having a high genetic risk in younger men whereas detection at a later age would come with a worse prognosis, is one potential explanation. The youngest age group, in which no differences in outcomes between different PRS groups were observed, in fact had the highest percentage of men with curative treatment (75% in the MDCS and 95% in the HPFS) (Supplementary Table 8). Crosswise cohort comparisons may also point to some role of early detection and treatment (men in the HPFS had earlier access to PSA testing and higher proportions of curative treatment), although it should be noted that other factors are also likely to account for the differences in risk observed between the cohorts which was higher for men in the MDCS throughout all ages.

Another plausible explanation for the results in the youngest age group is collider stratification bias, which is a type of selection bias resulting from only including cases in the analysis^23,24^. This bias may arise in case-only analyses if the risk factor of interest is also a risk factor for the disease itself (which is the scenario for PRSs of disease susceptibility), and if other risk factors that influence both the disease and survival exists and are not fully accounted for (such as other inherited genetic or environmental factors, and their interactions), making these factors “collide” in causing prostate cancer (a directed acyclic graph (DAG) is presented in Supplementary Fig. 1). This could induce bias and counter-intuitive associations, where for example an established risk factor shows no association or appears to be protective in a selected sample, sometimes referred to as a paradox^12,13^. An intuitive way to think about this bias is to consider a scenario in which the factor of interest is an established risk factor both for earlier disease onset and for earlier death. If a man has an early onset prostate cancer but without having this particular risk factor, this may indicate the presence of another, more harmful, risk factor^25^. Simple tabulation may support this: in addition to the PRS, both the proportion of smokers and the proportion of men with a family history of cancer was the highest in the youngest age group (Supplementary Table 8). Our previous work points to that only risk factors for disease onset that also have a strong link to survival, such as the presence of two genetic risk factors, may show a clear association in case-only survival analysis^26^.

Due to selection processes over the age span, multiple colliders may be present, as also highlighted by others^27^. Our results for the oldest age group, which were generally weaker than for the middle age group, may to some extent resemble a situation referred to as depletion of the susceptibles^28,29^. Intuitively, if we consider the same scenario as previously, those at highest risk have likely been diagnosed at an earlier age and the remaining population has been depleted of the most susceptible. Men with the risk factor of interest in this remaining population may have other protective factors and be particularly resistant, similar to conditioning the analysis on survival time up to a certain time point^30^. The impact of collider bias stratification may be the least in the middle age group because potentially less strongly influenced by the presence of other strong risk factors (such as genetic risk not captured by the PRS), and less affected by depletion of the susceptible and competing events. Our analysis further suggests that when comparing more extreme groups of the PRS, collider stratification bias may be more of a concern (Supplementary Table 1).

While the HRs of 1.21 to 1.25 appear to reflect properties of the sample rather than individual risks, which may be a consequence of collider bias^31,32^, further explanations may exist or coexist. The PRS is an aggregate of many different genetic factors, and studies of PRSs are particularly prone to bias^23,24^. The way the PRS was constructed may be relevant; the PRS may include risk variants mostly relevant for the middle age groups which are best represented in the underlying GWAS. Further, the PRS appears to identify both men with indolent and high risk cancers, diluting potential effects^33^. Lead-time bias may be a further issue, where a longer observed survival time due to potential earlier detection among men with a high PRS can bias estimates towards the null, presumably better controlled for when excluding variants related to PSA levels. Overall, these biases and issues necessitate cautious interpretation of findings from case-only analyses of PRS for prostate cancer susceptibility, which may be further complicated by heterogeneity in prostate cancer diagnosis as well as in cancer treatment.

Nevertheless, other studies have also indicated that genetic variability captured by in particular the 400-variant PRS could have a relationship with the risk of developing more aggressive prostate cancer^3,34^ or upgrading^35^, including from the large genetics consortium PRACTICAL^3^. In our study, the full cohort analysis may provide the most reliable indication, given that such an analysis is not affected to the same extent by the above-described biases. In both the MDCS and the HPFS, the association between the PRS (all versions) and prostate cancer mortality was stronger than the association between the PRS and prostate cancer incidence, a pattern that is not expected for a risk factor that influences incidence alone (under the assumption that the estimated HRs are fully comparable). Of note, a biomarker with such a pattern has previously been put forward as being useful for detecting men at high risk of dying from prostate cancer while reducing overdiagnosis of non-aggressive prostate cancers^36,37^. Yet, for population risk stratification and screening, a PRS that provides the strongest risk stratification for mortality may be preferable since it can capture more men at risk, in particular if combined with other biomarkers or tests to reduce overdiagnosis. In our analyses, the 451-variant PRS provided the strongest risk stratification for mortality, although the other versions showed a stronger association with survival, potentially indicating different clinical utility.

The analysis of individual variants further supports that variants within the PRS may have a link to the development of more aggressive disease, and in addition help understand the relationship with incidence, mortality, and survival in this setting. Nearly all of the 16 variants that remained after filtering by *p*-value had a pattern of a stronger association for mortality than incidence, and with similar directions of association for survival. A less strong association with incidence was also related to a stronger association in case-only survival analyses, which matches with theoretical reasoning around collider bias: factors with no or only a weak link to disease onset are less likely to “collide” in causing prostate cancer. This may also help explain why a longevity PRS, not specific to prostate cancer, showed an association with survival but not the prostate cancer PRS in a previous study^38^. In addition, several of the 16 identified variants were in regions that have previously been linked to worse prostate cancer-specific survival (4q24)^39^ or with a stronger association for high grade than low grade prostate cancer (8q24)^40^. Other variants alter protein structure within *SYTL1*, which is a gene that has been hypothesized to be involved in cancer progression^41^, *CHEK2* and *AN07*, both of which have been associated with aggressive prostate cancer^42,43^. Another of the identified variants is a regulatory variant of *CDKN2B-AS1*, encoding for long non-coding RNA linked to progression of other cancers and recently also prostate cancer^44,45^.

A few but important limitations of our study should be noted. The case-only analyses remain at risk for selection bias and with limited statistical power for comparisons of the youngest age group in particular. We cannot rule out the influence of other risk factors, other categorizations, or additional interactions (the presence of an interaction between risk factors has in simulations been shown to be a potent factor in introducing bias^46^). We have further mostly presented HRs, which we recognize have some potential problematic properties^30^ but which may be a minor concern in this case given the similarity to absolute risk comparisons. We recognize that the findings of our analysis may be specific to the data at hand and the calendar period under study, and are further influenced by late-age mortality (around half of all prostate cancer deaths occurred above the age of 80 years). Results may not be equally applicable to men diagnosed currently. Although the findings for the 16 individual genetic variants are largely in accordance with previous research, this part of the analysis is underpowered and should be considered exploratory with the need of further confirmation of findings. The strengths of this analysis include a rare opportunity to perform both full cohort and case-only analyses in independent cohorts of men, providing unique insight on inherited genetic risk factors, 20-year risk of dying of prostate cancer, and influences from potential biases.

## Conclusion

Our study indicates a link between already identified inherited genetic risk variants and worse prostate cancer outcomes, supported by both full cohort and case-only analyses in independent cohorts of men. In addition to providing strong stratification for prostate cancer risk, the 451-variants PRS, or versions of it, may also be informative for survival, at least in men not diagnosed early. The clinical relevance for men diagnosed in more recent calendar years needs further investigation. From a methodological perspective, our analysis demonstrates that case-only survival analyses of PRSs for prostate cancer susceptibility are sensitive to bias and potential misinterpretation of findings.

## Supplementary information

Transparent Peer Review file

Supplementary Information

Description of Additional Supplementary Files

Supplementary Data 1

Supplementary Data 2

Supplementary Data 3

Reporting summary

## Data Availability

The underlying cohort data are not publicly available due to privacy and ethical restrictions. Data can be made available for researchers through a project proposal for the Health Professionals Follow-up Study (https://hsph.harvard.edu/research/health-professionals/resources/for-external-collaborators/) and the Malmö Diet and Cancer Study (https://www.malmo-cohorts.lu.se/application-data-and-samples/applying-samples-mdc-and-mpp) according to the procedures described on the respective study websites. The estimates from the meta-analyses of all variants in the PRS (incidence, mortality, and survival) are provided in Supplementary Data 1. The source data for Fig. 1 are provided in Supplementary Data 2 and the source data for Fig. 2 in Supplementary Data 3. The 451-variant PRS is publicly available: https://www.pgscatalog.org/publication/PGP000488/.
