# Supplementary material for: Polygenic risk score and 20-year prostate cancer-specific mortality and survival
Source: Commun Med (Lond). 2026 Apr 24;6:243. doi: 10.1038/s43856-026-01603-9 (PMC13109360; doi:10.1038/s43856-026-01603-9)
Supplement: Supplementary file 2 — Supplementary Information [file 43856_2026_1603_MOESM2_ESM.pdf]

Supplementary Information

Polygenic Risk Score and 20-year Prostate Cancer-Specific Mortality and Survival

Plym et al.

Table of Contents

Supplementary Table 1. Quintile comparison of the different versions of the 451-variant PRS in relation to prostate cancer diagnosis (incidence) and prostate cancer death (mortality/survival), from meta-analyses of the MDCS and the HPFS. 2

Supplementary Table 2. Cohort-specific estimates of the different versions of the 451-variant PRS in relation to prostate cancer diagnosis (incidence) and prostate cancer death (mortality/survival) 3

Supplementary Table 3. Crosswise cohort comparisons of the PRSs filtered on p-value approach. 4

Supplementary Table 4. Cohort-specific estimates of different versions of the 451-variant PRS in relation to prostate cancer death (survival), stratified by age at diagnosis. 5

Supplementary Table 5. Combining the 400-variant PRS with family history of cancer in relation to prostate cancer diagnosis (incidence) and prostate cancer death (mortality/survival) 6

Supplementary Table 6. Combining the 400-variant PRS with family history of cancer in relation to prostate cancer death (survival), stratified by age at diagnosis. 7

Supplementary Table 7. Additional adjustment for lifestyle and clinical factors in the relationship between the 400-variant PRS and prostate cancer death (survival), stratified by age at diagnosis. 8

Supplementary Table 8. Characteristics of prostate cancer cases according to age at diagnosis. 9

Supplementary Figure 1. Directed acyclic graph (DAG) illustrating possible collider bias scenarios for the PRS. 10

Supplementary Contributor Information 11

Supplementary Acknowledgements 13

Supplementary Table 1. Quintile comparison of the different versions of the 451-variant PRS in relation to prostate cancer diagnosis (incidence) and prostate cancer death (mortality/survival), from meta-analyses of the MDCS and the HPFS.

	Incidence (full cohort)		Mortality (full cohort)		Survival (case-only)	
	Events/ pyrs	HR (95% CI) [a]	Events/ pyrs	HR (95% CI) [a]	Events/ pyrs	HR (95% CI) [b]
451-variants PRS						
0-20%	233/120111	0.37 (0.29-0.49)	38/128891	0.46 (0.31-0.69)	38/2347	1.16 (0.78-1.70)
20-40%	420/115327	0.70 (0.54-0.91)	51/125918	0.66 (0.46-0.94)	51/4303	0.94 (0.66-1.34)
40-60%	589/110414	1 (Ref.)	78/122388	1 (Ref.)	78/6165	1 (Ref.)
60-80%	800/107446	1.43 (1.12-1.83)	104/121731	1.35 (0.81-2.27)	104/8573	1.02 (0.75-1.40)
80-100%	1309/92025	2.70 (2.27-3.21)	173/110959	2.58 (1.97-3.38)	173/14482	1.09 (0.83-1.43)
400-variant PRS (excluding PSA variants)						
0-20%	271/118518	0.43 (0.37-0.51)	39/127691	0.56 (0.38-0.84)	39/2774	1.32 (0.89-1.97)
20-40%	461/114718	0.76 (0.66-0.86)	61/125659	0.91 (0.64-1.30)	61/4842	1.19 (0.84-1.69)
40-60%	582/109148	1 (Ref.)	65/120816	1 (Ref.)	65/6201	1 (Ref.)
60-80%	796/107944	1.39 (1.24-1.57)	107/122400	1.67 (1.23-2.28)	107/8383	1.29 (0.95-1.76)
80-100%	1241/94996	2.47 (2.22-2.76)	172/113319	2.86 (2.15-3.82)	172/13670	1.35 (1.01-1.79)
16-variant PRS for mortality						
0-20%	481/113291	0.73 (0.64-0.83)	38/124739	0.44 (0.30-0.64)	38/5301	0.59 (0.40-0.86)
20-40%	600/111440	0.93 (0.82-1.05)	66/123391	0.78 (0.57-1.08)	66/6237	0.87 (0.63-1.20)
40-60%	649/111699	1 (Ref.)	87/125195	1 (Ref.)	87/7069	1 (Ref.)
60-80%	736/105961	1.19 (1.06-1.34)	101/118977	1.22 (0.91-1.63)	101/7787	1.06 (0.74-1.53)
80-100%	885/102933	1.47 (1.31-1.64)	152/117585	1.89 (1.45-2.47)	152/9476	1.39 (1.06-1.80)

Abbreviations: CI, confidence interval; HPFS, Health Professionals Follow-up Study; HR, hazard ratio; MDCS, Malmo Diet and Cancer Cohort; PRS, polygenic risk score.
a. From models using age as the underlying timescale.
b. From models using time since diagnosis as the underlying timescale, with additional adjustment for age at diagnosis.

Supplementary Table 2. Cohort-specific estimates of the different versions of the 451-variant PRS in relation to prostate cancer diagnosis (incidence) and prostate cancer death (mortality/survival).

	Incidence (full cohort)		Mortality (full cohort)		Survival (case-only)	
	Events/pyrs	HR (95% CI) [a]	Events/pyrs	HR (95% CI) [a]	Events/pyrs	HR (95% CI) [b]
MDCS						
451-variants PRS						
0-50%	431/103027	1 (Ref.)	71/106751	1 (Ref.)	71/3725	1 (Ref.)
50-100%	1143/94270	2.95 (2.64-3.30)	217/104619	3.21 (2.46-4.20)	217/10351	1.24 (0.94-1.62)
400-variant PRS (excluding PSA variants)						
0-50%	456/102759	1 (Ref.)	74/106760	1 (Ref.)	74/4002	1 (Ref.)
50-100%	1118/94538	2.72 (2.43-3.03)	214/104610	3.03 (2.33-3.95)	214/10074	1.30 (1.00-1.69)
16-variant PRS for mortality						
0-50%	629/100658	1 (Ref.)	88/106232	1 (Ref.)	88/5575	1 (Ref.)
50-100%	945/96640	1.57 (1.42-1.74)	200/105138	2.32 (1.81-2.99)	200/8501	1.60 (1.25-2.06)
HPFS						
451-variants PRS						
0-50%	483/188422	1 (Ref.)	42/209889	1 (Ref.)	42/5710	1 (Ref.)
50-100%	1294/159604	3.11 (2.75-3.51)	114/188627	3.35 (2.33-4.81)	114/16084	1.17 (0.82-1.68)
400-variant PRS (excluding PSA variants)						
0-50%	551/184946	1 (Ref.)	48/206654	1 (Ref.)	48/6635	1 (Ref.)
50-100%	1226/163081	2.49 (2.21-2.80)	108/191861	2.60 (1.83-3.68)	108/15159	1.17 (0.83-1.65)
16-variant PRS for mortality						
0-50%	768/178791	1 (Ref.)	59/203119	1 (Ref.)	59/9488	1 (Ref.)
50-100%	1009/169236	1.37 (1.23-1.54)	97/195397	1.77 (1.27-2.47)	97/12307	1.29 (0.93-1.78)

Abbreviations: CI, confidence interval; HPFS, Health Professionals Follow-up Study; HR, hazard ratio; MDCS, Malmö Diet and Cancer Study; PRS, polygenic risk score; pyrs, person-years; Ref., reference;

a. From models using age as the underlying timescale.

b. From models using time since diagnosis as the underlying timescale, with additional adjustment for age at diagnosis.

Supplementary Table 3. Crosswise cohort comparisons of the PRSs filtered on p-value approach.

	Survival (case-only)							
	All ages		Age < 65 years		Age 65-74 years		Age ≥ 75 years	
	Events/pyrs	HR (95% CI) [a]	Events/pyrs	HR (95% CI) [a]	Events/pyrs	HR (95% CI) [a]	Events/pyrs	HR (95% CI) [a]
MDCS								
PRS for mortality based on p-values from HPFS and PHS only								
0-50%	127/6359	1 (Ref.)	17/1475	1 (Ref.)	58/3562	1 (Ref.)	52/1322	1 (Ref.)
50-100%	161/7716	1.10 (0.87-1.39)	18/1936	0.82 (0.42-1.59)	77/4262	1.11 (0.79-1.56)	66/1518	1.10 (0.77-1.58)
HPFS								
PRS for mortality based on p-values from MDCS and PHS only								
0-50%	64/9403	1 (Ref.)	10/2390	1 (Ref.)	18/4846	1 (Ref.)	36/2168	1 (Ref.)
50-100%	92/12391	1.07 (0.78-1.47)	14/3371	0.99 (0.44-2.23)	39/6088	1.72 (0.99-3.01)	39/2932	0.80 (0.51-1.26)

Abbreviations: CI, confidence interval; HPFS, Health Professionals Follow-up Study; HR, hazard ratio; MDCS, Malmö Diet and Cancer Study; PHS, Physicians' Health Study; PRS, polygenic risk score; pyrs, person-years; Ref., reference;
a. From models stratified by age at diagnosis.

Supplementary Table 4. Cohort-specific estimates of different versions of the 451-variant PRS in relation to prostate cancer death (survival), stratified by age at diagnosis.

	Survival (case-only)					
	Age < 65 years		Age 65-74 years		Age ≥ 75 years	
	Events/pyrs	HR (95% CI) [a]	Events/pyrs	HR (95% CI) [a]	Events/pyrs	HR (95% CI) [a]
MDCS						
451-variants PRS						
0-50%	10/656	1 (Ref.)	29/2196	1 (Ref.)	32/873	1 (Ref.)
50-100%	25/2756	0.61 (0.29-1.28)	106/5628	1.44 (0.95-2.17)	86/1967	1.19 (0.79-1.78)
400-variant PRS (excluding PSA variants)						
0-50%	9/669	1 (Ref.)	27/2415	1 (Ref.)	38/918	1 (Ref.)
50-100%	26/2742	0.72 (0.34-1.53)	108/5410	1.82 (1.19-2.78)	80/1922	1.00 (0.68-1.47)
16-variant PRS for mortality						
0-50%	13/1233	1 (Ref.)	39/3122	1 (Ref.)	36/1220	1 (Ref.)
50-100%	22/2178	0.96 (0.48-1.91)	96/4702	1.64 (1.13-2.38)	82/1620	1.71 (1.15-2.53)
HPFS						
451-variants PRS						
0-50%	7/1231	1 (Ref.)	9/2646	1 (Ref.)	26/1833	1 (Ref.)
50-100%	17/4530	0.65 (0.27-1.58)	48/8287	1.71 (0.84-3.49)	49/3267	1.06 (0.66-1.70)
400-variant PRS (excluding PSA variants)						
0-50%	8/1456	1 (Ref.)	12/3144	1 (Ref.)	28/2035	1 (Ref.)
50-100%	16/4305	0.67 (0.29-1.57)	45/7790	1.51 (0.80-2.86)	47/3065	1.12 (0.70-1.79)
16-variant PRS for mortality						
0-50%	11/2352	1 (Ref.)	17/4882	1 (Ref.)	31/2254	1 (Ref.)
50-100%	13/3409	0.82 (0.37-1.82)	40/6051	1.90 (1.08-3.35)	44/2846	1.11 (0.70-1.76)

Abbreviations: CI, confidence interval; HPFS, Health Professionals Follow-up Study; HR, hazard ratio; MDCS, Malmö Diet and Cancer Study; PRS, polygenic risk score; pyrs, person-years; Ref., reference;

a. From models stratified by age at diagnosis.

Supplementary Table 5. Combining the 400-variant PRS with family history of cancer in relation to prostate cancer diagnosis (incidence) and prostate cancer death (mortality/survival).

	Incidence (full cohort)		Mortality (full cohort)		Survival (case-only)	
	Events/pyrs	HR (95% CI) [a]	Events/pyrs	HR (95% CI) [a]	Events/pyrs	HR (95% CI) [b]
MDCS						
400-variant PRS (excluding PSA variants)						
0-50% and no FH [c]	361/80729	1 (Ref.)	58/83931	1 (Ref.)	58/3203	1 (Ref.)
50-100% and no FH [c]	832/74965	2.53 (2.24-2.86)	153/82421	2.78 (2.05-3.76)	153/7457	1.25 (0.93-1.69)
FH	381/41603	2.08 (1.80-2.40)	77/45018	2.57 (1.83-3.61)	77/3416	1.51 (1.07-2.12)
HPFS						
400-variant PRS (excluding PSA variants)						
0-50% and no FH [c]	458/163260	1 (Ref.)	38/182161	1 (Ref.)	38/5513	1 (Ref.)
50-100% and no FH [c]	961/138349	2.44 (2.14-2.78)	83/161663	2.62 (1.77-3.88)	83/11745	1.22 (0.83-1.80)
FH	358/46418	2.75 (2.32-3.25)	35/54691	3.19 (1.99-5.10)	35/4536	1.41 (0.89-2.23)
Meta-analysis						
400-variant PRS (excluding PSA variants)						
0-50% and no FH [c]		1 (Ref.)		1 (Ref.)		1 (Ref.)
50-100% and no FH [c]		2.49 (2.27-2.72)		2.72 (2.14-3.45)		1.24 (0.98-1.57)
FH		2.38 (1.81-3.13)		2.77 (2.10-3.65)		1.47 (1.12-1.93)

Abbreviations: CI, confidence interval; FH, family history; HPFS, Health Professionals Follow-up Study; HR, hazard ratio; MDCS, Malmö Diet and Cancer Study; PRS, polygenic risk score; pyrs, person-years; Ref., reference;

a. From models using age as the underlying timescale.

b. From models using time since diagnosis as the underlying timescale, with additional adjustment for age at diagnosis.

c. Family history indicates a first-degree family history of prostate cancer (HPFS) or any history of cancer in the father (MDCS).

Supplementary Table 6. Combining the 400-variant PRS with family history of cancer in relation to prostate cancer death (survival), stratified by age at diagnosis.

	Survival (case-only)					
	Age < 65 years		Age 65-74 years		Age ≥ 75 years	
	Events/pyrs	HR (95% CI) [a]	Events/pyrs	HR (95% CI) [a]	Events/pyrs	HR (95% CI) [a]
MDCS						
400-variant PRS (excluding PSA variants)						
0-50% and no FH[b]	8/517	1 (Ref.)	18/1954	1 (Ref.)	32/732	1 (Ref.)
50-100% and no FH[b]	17/1840	0.60 (0.26-1.38)	75/4075	2.06 (1.23-3.44)	61/1542	0.91 (0.59-1.39)
FH	10/1054	0.60 (0.24-1.52)	42/1795	2.69 (1.54-4.68)	25/566	1.00 (0.60-1.69)
HPFS						
400-variant PRS (excluding PSA variants)						
0-50% and no FH[b]	6/1195	1 (Ref.)	8/2561	1 (Ref.)	24/1757	1 (Ref.)
50-100% and no FH[b]	8/3140	0.51 (0.18-1.46)	37/6169	1.92 (0.90-4.13)	38/2435	1.16 (0.69-1.93)
FH	10/1426	1.39 (0.51-3.83)	12/2203	1.75 (0.71-4.28)	13/907	1.05 (0.54-2.07)
Meta-analysis						
400-variant PRS (excluding PSA variants)						
0-50% and no FH[b]		1 (Ref.)		1 (Ref.)		1 (Ref.)
50-100% and no FH[b]		0.56 (0.29-1.08)		2.01 (1.31-3.09)		1.00 (0.72-1.39)
FH		0.89 (0.39-2.03)		2.39 (1.49-3.82)		1.02 (0.68-1.55)

Abbreviations: CI, confidence interval; FH, family history; HPFS, Health Professionals Follow-up Study; HR, hazard ratio; MDCS, Malmö Diet and Cancer Study; PRS, polygenic risk score; pyrs, person-years; Ref., reference;

a. From models stratified by age at diagnosis.

b. Family history indicates a first-degree family history of prostate cancer (HPFS) or any history of cancer in the father (MDCS).

Supplementary Table 7. Additional adjustment for lifestyle and clinical factors in the relationship between the 400-variant PRS and prostate cancer death (survival), stratified by age at diagnosis.

	Survival (case-only)					
	Age < 65 years		Age 65-74 years		Age ≥ 75 years	
	Events/pyrs	HR (95% CI) [a]	Events/pyrs	HR (95% CI) [a]	Events/pyrs	HR (95% CI) [a]
MDCS						
400-variant PRS (excluding PSA variants)						
0-50%	9/669	1 (Ref.)	27/2415	1 (Ref.)	38/918	1 (Ref.)
50-100%	26/2742	0.71 (0.33-1.53)	108/5410	1.90 (1.24-2.92)	80/1922	0.90 (0.61-1.33)
HPFS						
400-variant PRS (excluding PSA variants)						
0-50%	8/1456	1 (Ref.)	12/3144	1 (Ref.)	28/2035	1 (Ref.)
50-100%	16/4305	0.66 (0.27-1.61)	45/7790	1.65 (0.86-3.18)	47/3065	0.95 (0.59-1.54)
Meta-analysis						
400-variant PRS (excluding PSA variants)						
0-50%		1 (Ref.)		1 (Ref.)		1 (Ref.)
50-100%		0.69 (0.38-1.23)		1.82 (1.27-2.61)		0.92 (0.68-1.24)

Abbreviations: CI, confidence interval; HPFS, Health Professionals Follow-up Study; HR, hazard ratio; MDCS, Malmö Diet and Cancer Study; PRS, polygenic risk score; pyrs, person-years; Ref., reference;

a. From models stratified by age at diagnosis, with additional adjustment for smoking, body mass index, calendar year of diagnosis, NCCN risk categories and primary treatment.

Supplementary Table 8. Characteristics of prostate cancer cases according to age at diagnosis.

	MDCS			HPFS		
	Age 50-64 (n = 247)	Age 65-74 (n = 803)	Age ≥75 (n = 524)	Age 50-64 (n = 376)	Age 65-74 (n = 842)	Age ≥75 (n = 559)
451-variant PRS, n (%)						
0-20%	7 (2.8)	52 (6.5)	53 (10.1)	21 (5.6)	50 (5.9)	50 (8.9)
20-40%	24 (9.7)	102 (12.7)	75 (14.3)	40 (10.6)	90 (10.7)	89 (15.9)
40-60%	36 (14.6)	122 (15.2)	93 (17.7)	58 (15.4)	160 (19.0)	120 (21.5)
60-80%	51 (20.6)	212 (26.4)	116 (22.1)	80 (21.3)	234 (27.8)	107 (19.1)
80-100%	129 (52.2)	315 (39.2)	187 (35.7)	177 (47.1)	308 (36.6)	193 (34.5)
Year of diagnosis, n (%)						
< 2000	79 (32.0)	183 (22.8)	15 (2.9)	109 (29.0)	241 (28.6)	106 (19.0)
2000-2004	105 (42.5)	259 (32.3)	104 (19.8)	154 (41.0)	294 (34.9)	185 (33.1)
2005-2009	62 (25.1)	157 (19.6)	112 (21.4)	108 (28.7)	217 (25.8)	175 (31.3)
≥ 2010	1 (0.4)	204 (25.4)	293 (55.9)	5 (1.3)	90 (10.7)	93 (16.6)
NCCN risk groups, n (%)						
Low	104 (47.5)	227 (30.8)	62 (13.6)	156 (42.4)	308 (38.2)	148 (29.1)
Intermediate	56 (25.6)	219 (29.7)	102 (22.3)	171 (46.5)	363 (45.0)	212 (41.7)
High	42 (19.2)	226 (30.6)	211 (46.2)	38 (10.3)	129 (16.0)	128 (25.2)
Distant metastasis	17 (7.8)	66 (8.9)	82 (17.9)	3 (0.8)	7 (0.9)	20 (3.9)
Missing	28	65	67	8	35	51
Treatment strategy, n (%)						
Conservative	35 (15.9)	205 (27.6)	133 (28.5)	11 (3.0)	54 (6.8)	106 (21.1)
Curative	164 (74.5)	374 (50.3)	71 (15.2)	349 (95.4)	711 (88.9)	297 (59.2)
Hormone therapy/other treatment	21 (9.5)	164 (22.1)	263 (56.3)	6 (1.6)	35 (4.4)	99 (19.7)
Missing	27	60	57	10	42	57
Family history [a]	75 (30.4)	197 (24.5)	109 (20.8)	95 (25.3)	171 (20.3)	92 (16.5)
Current smoker or quit <10 years	100 (40.5)	292 (36.4)	155 (29.6)	41 (10.9)	80 (9.5)	49 (8.8)
BMI ≥ 30 kg/m^2	29 (11.7)	97 (12.1)	51 (9.7)	41 (10.9)	94 (11.2)	51 (9.1)

Abbreviations: BMI, body mass index; HPFS, Health Professionals Follow-up Study; IQR, interquartile range; NCCN, National Comprehensive Cancer Network; MDCS, Malmö Diet and Cancer Study; PRS, polygenic risk score.
a. Family history indicates a first-degree family history of prostate cancer (HPFS) or any history of cancer in the father (MDCS).

Supplementary Figure 1. Directed acyclic graph (DAG) illustrating possible collider bias scenarios for the PRS. A) illustrates a general DAG when conditioning on prostate cancer; B) includes a second collider (here earlier diagnosis of prostate cancer; but could also be death before age a_2). U represents unmeasured common causes of prostate cancer and prostate cancer death. The box indicates selection. The dashed line represents an induced association because of the selection.

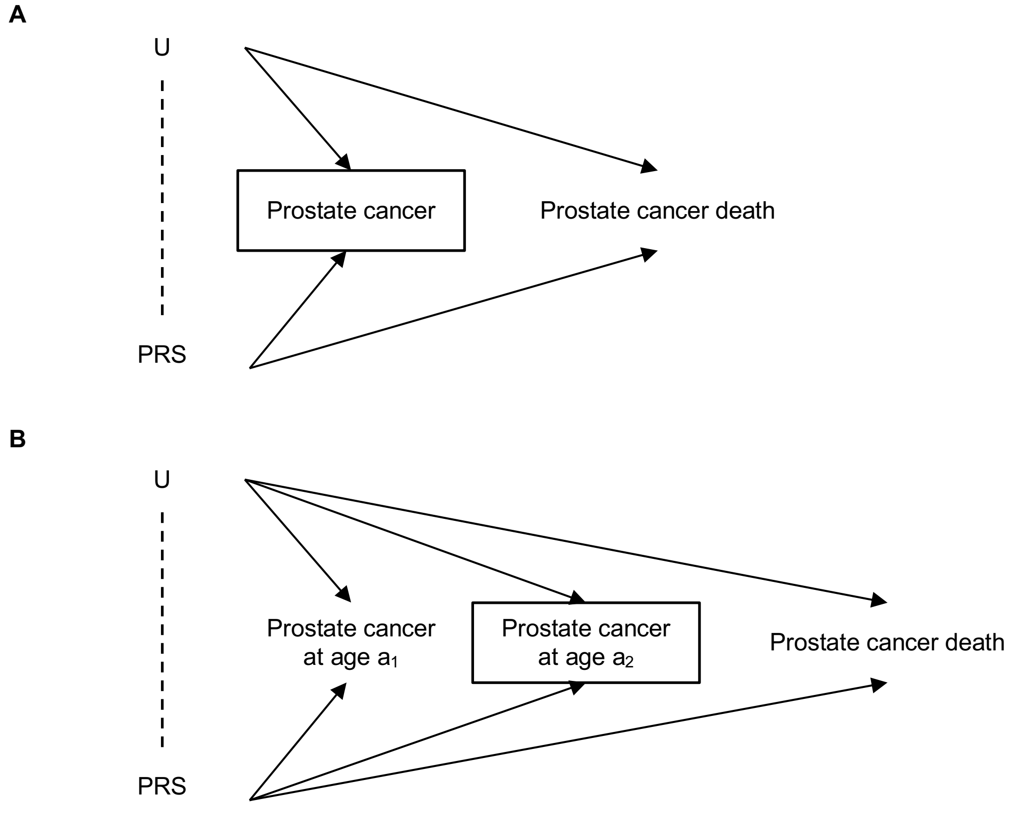

Supplementary Contributor Information

Regeneron Genetics Center members

RGC Management & Leadership Team
Gonçalo Abecasis, D. Phil., Adolfo Ferrando, M.D., Ph.D., Aris Baras, M.D., Michael Cantor, M.D., Giovanni Coppola, M.D., Andrew Deubler, M.P.S., Aris Economides, Ph.D., Luca A Lotta, M.D., Ph.D., John D Overton, Ph.D., Jeffrey G Reid, Ph.D., Alan Shuldiner, M.D., Katherine Siminovitch, M.D.

Contribution: All authors contributed to securing funding, study design and oversight. All authors reviewed the final version of the manuscript.

Sequencing & Lab Operations
John D Overton, Ph.D., Christina Beechert, Erin D Brian, Laura M Cremona, Ph.D., Hang Du, Caitlin Forsythe, M.S., Zhenhua Gu, M.S., Kristy Guevara, M.S., Michael Lattari, Alexander Lopez, M.S., Kia Manoochehri, Prathyusha Challa, M.S., Manasi Pradhan, M.S., Raymond Reynoso, Ricardo Schiavo, Maria Sotiropoulos Padilla, M.S., Chenggu Wang, M.S., Sarah E Wolf, M.S.

Contribution: Performed and are responsible for sample genotyping and exome sequencing, conceived and are responsible for laboratory automation, and responsible for sample tracking and the library information management system.

Clinical Informatics
Michael Cantor M.D. , Amelia Averitt, Ph.D., Nilanjana Banerjee, Ph.D., Dadong Li, Ph.D., Sameer Malhotra, M.D., Justin Mower, Ph.D., Mudasar Sarwar, Deepika Sharma, Ph.D., Jeffrey C Staples, Ph.D., Jay Sundaram, Sean Yu, Ph.D., Aaron Zhang, Ph.D.

Contribution: Development and validation of clinical phenotypes used to identify study participants and (when applicable) controls.

Genome Informatics & Data Engineering
Jeffrey G Reid, Ph.D., Mona Nafde, M.S., George Mitra, Sujit Gokhale, Andrew Bunyea, Janice Clauer, M.S., MBA, Krishna Pawan Punuru, M.S., Sanjay Sreeram, Gisu Eom, Sujit Gokhale, Benjamin Sultan, M.S., Rouel Lanche, Vrushali Mahajan, Eliot Austin, Koteswararao Makkena, M.S., Sean O'Keeffe, Ph.D., Razvan Panea, Ph.D., Tommy Polanco, Ayesha Rasool, M.S., William Salerno, Ph.D., Xiaodong Bai, Ph.D., Lance Zhang, M.S., Boris Boutkov, Ph.D., Evan Edelstein, Alexander Gorovits, Ph.D., Ju Guan, Ph.D., Lukas Habegger, Ph.D., Alicia Hawes, Olga Krasheninina, M.S., Samantha Zarate, Ph.D., Adam J Mansfield, Evan K Maxwell, Ph.D., Suganthi Balasubramanian, Ph.D., Suying Bao, Ph.D., Kathie Sun, Ph.D., Chuanyi Zhang, Ph.D.

Contribution: Performed and are responsible for analysis needed to produce exome and genotype data, provided compute infrastructure development and operational support, provided variant and gene annotations and their functional interpretation of variants, and conceived and are responsible for creating, developing, and deploying analysis platforms and computational methods for analyzing genomic data.

Analytical Genetics and Data Science
Gonçalo Abecasis, D. Phil., Manuel Allen Revez Ferreira, Ph.D., Joshua Backman, Ph.D., Kathy Burch, Ph.D., Adrian Campos, Ph.D., Lei Chen, Ph.D., Sam Choi, Ph.D., Amy Damask, Ph.D., Liron Ganel, Ph.D., Sheila Gaynor, Ph.D., Benjamin Geraghty, Ph.D., Akropravo Ghosh, M.S., Salvador Romero Martinez, Christopher Gillies, Ph.D., Lauren Gurski, Joseph Herman, D. Phil., Eric Jorgenson, Ph.D., Tyler Joseph, Ph.D., Michael Kessler, Ph.D., Jack Kosmicki, Ph.D., Nan Lin, Ph.D., Adam Locke, Ph.D., Priyanka Nakka, Ph.D., Jonathan Marchini, Ph.D., Karl

Landheer, Ph.D., Olivier Delaneau, Ph.D., Maya Ghoussaini, Ph.D., Anthony Marcketta, M.S., Joelle Mbatchou, Ph.D., Arden Moscati, Ph.D., Aditeya Pandey, Ph.D., Anita Pandit, M.S., Charles Paulding, Ph.D., Jonathan Ross, Carlo Sidore, Ph.D., Eli Stahl, Ph.D., Maria Suciu, Ph.D., Timothy Thornton, Ph.D., Peter VandeHaar, M.S., Sailaja Vedantam, Ph.D., Scott Vrieze, Ph.D., Jingning Zhang, Ph.D., Rujin Wang, Ph.D., Kuan-Han Wu, Ph.D., Bin Ye, Ph.D., Blair Zhang, Ph.D., Andrey Ziyatdinov, Ph.D., Yuxin Zou, Ph.D., Olivier Delaneau, Ph.D., Maya Ghoussaini, Ph.D., Jingning Zhang, Ph.D.

Contribution: Development of statistical analysis plans. QC of genotype and phenotype files and generation of analysis ready datasets. Development of statistical genetics pipelines and tools and use thereof in generation of the association results. QC, review and interpretation of results. Generation and formatting of results for manuscript figures.

Therapeutic Area Genetics
Adolfo Ferrando, M.D., Ph.D., Giovanni Coppola, M.D., Luca A Lotta, M.D., Ph.D., Alan Shuldiner, M.D., Katherine Siminovitch, M.D., Brian Hobbs, M.D., Jon Silver, Ph.D., William Palmer, Ph.D., Rita Guerreiro, Ph.D., Amit Joshi, Ph.D., Antoine Baldassari, Ph.D., Cristen Willer, D. Phil., Sarah Graham, Ph.D., Ernst Mayerhofer, M.D., Jonas Bille Nielsen, Ph.D., Mary Hass, Ph.D., Niek Verwei, Ph.D.1j, George Hindy, Ph.D., Jonas Bovijn, M.D., Tanima De, Ph.D., Parsa Akbari, Ph.D., Luanluan Sun, Ph.D., Olukayode Sosina, Ph.D., Arthur Gilly, Ph.D., Peter Dombos, Ph.D., Juan Rodriguez-Flores, Ph.D., Moeen Riaz, Ph.D., Manav Kapoor, Ph.D., Gannie Tzoneva, Ph.D., Momodou W Jallow, Ph.D., Anna Alkelai, Ph.D., Giovanni Coppola, M.D., Ariane Ayer, Veera Rajagopal, M.D., Sahar Gelfman, Ph.D., Vijay Kumar, Ph.D., Jacqueline Otto, Ph.D., Neel Parikshak, M.D., Aysegul Guvenek, Ph.D., Jose Bras, Ph.D., Silvia Alvarez, Ph.D., Jessie Brown, Ph.D., Jing He, Ph.D., Hossein Khiabanian, Ph.D.

Contribution: Development of study design and analysis plans. Development and QC of phenotype definitions. QC, review, and interpretation of association results.

Research Program Management & Strategic Initiatives
Lyndon J Mitnaul, Ph.D., Marcus B Jones, Ph.D., Esteban Chen, M.S., Michelle G LeBlanc, Ph.D., Jason Mighty, Ph.D., Nirupama Nishtala, Ph.D., Nadia Rana, Ph.D., Jennifer Rico-Varela, Ph.D., Jaimee Hernandez.

Contribution: Contributed to the management and coordination of all research activities, planning and execution, managed the review of the project.

Senior Partnerships & Business Operations
Alison Fenney, Ph.D., MBA, Jody Hankins, Ph.D., MBA, Samuel Hart, J.D.

Contribution: Contributed to the management, planning, execution, and negotiation of new and existing agreements.

Business Operations & Administrative Coordinators
Ann Perez-Beals, Gina Solari, Jaimee Hernandez, Johannie Rivera-Picart, Michelle Pagan, Sunilbe Siceron.

Contribution: coordinate all administrative activities with internal stakeholders and external collaborators.

Supplementary Acknowledgements

NPCR

This project was made possible by the continuous work of the Swedish National Prostate Cancer Register (NPCR) steering group: Ingela Franck Lissbrant, Johan Styrke, David Robinson, Johan Stranne, Jon Kindblom, Camilla Thellenberg, Andreas Josefsson, Ingrida Verbiene, Hampus Nugin, Stefan Carlsson, Anna Kristiansen, Mats Andén, Thomas Jiborn, Olof Ståhl, Olof Akre, Per Fransson, Eva Johansson, Magnus Törnblom, Fredrik Jäderling, Marie Hjälm Eriksson, Lotta Renström, Jonas Hugosson, Ola Bratt, Maria Nyberg, Fredrik Sandin, Camilla Byström, Muia Brus, Mats Lambe, Anna Hedström, Nina Hageman, Christofer Lagerros, Hans Joelsson and Gert Malmberg.

HPFS

The authors would like to acknowledge the contribution to this study from central cancer registries supported through the Centers for Disease Control and Prevention's National Program of Cancer Registries (NPCR) and/or the National Cancer Institute's Surveillance, Epidemiology, and End Results (SEER) Program. Central registries may also be supported by state agencies, universities, and cancer centers. Participating central cancer registries include the following: Alabama, Alaska, Arizona, Arkansas, California, Delaware, Colorado, Connecticut, Florida, Georgia, Hawaii, Idaho, Indiana, Iowa, Kentucky, Louisiana, Maine, Maryland, Massachusetts, Michigan, Mississippi, Montana, Nebraska, Nevada, New Hampshire, New Jersey, New Mexico, New York, North Carolina, North Dakota, Ohio, Oklahoma, Oregon, Pennsylvania, Puerto Rico, Rhode Island, Seattle SEER Registry, South Carolina, Tennessee, Texas, Utah, Virginia, West Virginia, Wyoming.

CRUK and PRACTICAL consortium

This work was supported by the Canadian Institutes of Health Research, European Commission's Seventh Framework Programme grant agreement n° 223175 (HEALTH-F2-2009-223175), Cancer Research UK Grants C5047/A7357, C1287/A10118, C1287/A16563, C5047/A3354, C5047/A10692, C16913/A6135, and The National Institute of Health (NIH) Cancer Post-Cancer GWAS initiative grant: No. 1 U19 CA 148537-01 (the GAME-ON initiative).

We would also like to thank the following for funding support: The Institute of Cancer Research and The Everyman Campaign, The Prostate Cancer Research Foundation, Prostate Research Campaign UK (now PCUK), The Orchid Cancer Appeal, Rosetrees Trust, The National Cancer Research Network UK, The National Cancer Research Institute (NCRI) UK. We are grateful for support of NIHR funding to the NIHR Biomedical Research Centre at The Institute of Cancer Research, The Royal Marsden NHS Foundation Trust, and Manchester NIHR Biomedical Research Centre. The Prostate Cancer Program of Cancer Council Victoria also acknowledge grant support from The National Health and Medical Research Council, Australia (126402, 209057, 251533, , 396414, 450104, 504700, 504702, 504715, 623204, 940394, 614296,), VicHealth, Cancer Council Victoria, The Prostate Cancer Foundation of Australia, The Whitten Foundation, PricewaterhouseCoopers, and Tattersall's. EAO, DMK, and EMK acknowledge the Intramural Program of the National Human Genome Research Institute for their support.

Genotyping of the OncoArray was funded by the US National Institutes of Health (NIH) [U19 CA 148537 for ELucidating Loci Involved in Prostate cancer SuscEptibility (ELLIPSE) project and X01HG007492 to the Center for Inherited Disease Research (CIDR) under contract number HHSN268201200008I]. Additional analytic support was provided by NIH NCI U01 CA188392 (PI: Schumacher).

Research reported in this publication also received support from the National Cancer Institute of the National Institutes of Health under Award Numbers U10 CA37429 (CD Blanke), and UM1 CA182883 (CM Tangen/IM Thompson). The content is solely the responsibility of the authors and does not necessarily represent the official views of the National Institutes of Health.

Funding for the iCOGS infrastructure came from: the European Community's Seventh Framework Programme under grant agreement n° 223175 (HEALTH-F2-2009-223175) (COGS), Cancer Research UK (C1287/A10118, C1287/A 10710, C12292/A11174, C1281/A12014, C5047/A8384, C5047/A15007, C5047/A10692, C8197/A16565), the

National Institutes of Health (CA128978) and Post-Cancer GWAS initiative (1U19 CA148537, 1U19 CA148065 and 1U19 CA148112 - the GAME-ON initiative), the Department of Defence (W81XWH-10-1-0341), the Canadian Institutes of Health Research (CIHR) for the CIHR Team in Familial Risks of Breast Cancer, Komen Foundation for the Cure, the Breast Cancer Research Foundation, and the Ovarian Cancer Research Fund.

BPC3

The BPC3 was supported by the U.S. National Institutes of Health, National Cancer Institute (cooperative agreements U01-CA98233 to D.J.H., U01-CA98710 to S.M.G., U01-CA98216 to E.R., and U01-CA98758 to B.E.H., and Intramural Research Program of NIH/National Cancer Institute, Division of Cancer Epidemiology and Genetics).

CAPS

CAPS GWAS study was supported by the Cancer Risk Prediction Center (CRisP; www.crispcenter.org), a Linneus Centre (Contract ID 70867902) financed by the Swedish Research Council, (grant no K2010-70X-20430-04-3), the Swedish Cancer Foundation (grant no 09-0677), the Hedlund Foundation, the Soederberg Foundation, the Enqvist Foundation, ALF funds from the Stockholm County Council. Stiftelsen Johanna Hagstrand och Sigfrid Linner's Minne, Karlsson's Fund for urological and surgical research.

PEGASUS

PEGASUS was supported by the Intramural Research Program, Division of Cancer Epidemiology and Genetics, National Cancer Institute, National Institutes of Health
