# Supplementary material for: Polygenic risk score and 20-year prostate cancer-specific mortality and survival
Source: Commun Med (Lond). 2026 Apr 24;6:243. doi: 10.1038/s43856-026-01603-9 (PMC13109360; doi:10.1038/s43856-026-01603-9)
Supplement: Supplementary file 3 — Description of Additional Supplementary Files [file 43856_2026_1603_MOESM3_ESM.pdf]

Description of Additional Supplementary Files

Polygenic Risk Score and 20-year Prostate Cancer-Specific Mortality and Survival

Plym et al.

Supplementary Data 1

The estimates (log hazard ratios) from the meta-analyses of all variants in the PRS (incidence, mortality, and survival). See further explanations in sheet 2 of the file.

Supplementary Data 2

Source data for Figure 1. Included variables: CIF, specifies subgroup; P, probability of prostate cancer death; time, time since diagnosis; var, variance; lower, lower 95% confidence interval; upper, upper 95% confidence interval; cohort, specifies the cohort; prs, specifies the prs.

Supplementary Data 3

Source data for Figure 2. Included variables: CIF, specifies subgroup; P, probability of prostate cancer death; time, time since diagnosis; var, variance; lower, lower 95% confidence interval; upper, upper 95% confidence interval; cohort, specifies the cohort; group, specifies the age groups.
