# Supplementary material for: Polygenic risk score and 20-year prostate cancer-specific mortality and survival
Source: Commun Med (Lond). 2026 Apr 24;6:243. doi: 10.1038/s43856-026-01603-9 (PMC13109360; doi:10.1038/s43856-026-01603-9)
Supplement: Supplementary file 1 — Transparent Peer Review file [file 43856_2026_1603_MOESM1_ESM.pdf]

nature portfolio

Peer Review File

Polygenic Risk Score and 20-year Prostate Cancer-Specific Mortality and Survival

Corresponding Author: Dr Anna Plym

This file contains all reviewer reports in order by version, followed by all author rebuttals in order by version.

Version 0:

Reviewer comments:

Reviewer #1

(Remarks to the Author)
The authors have addressed the points that were highlighted.

Reviewer #2

(Remarks to the Author)
All comments seem to have been adequately addressed.

Reviewer #3

(Remarks to the Author)
I co-reviewed this manuscript with one of the reviewers who provided the listed reports. This is part of the Communications Medicine initiative to facilitate training in peer review and to provide appropriate recognition for Early Career Researchers who co-review manuscripts.

Open Access This Peer Review File is licensed under a Creative Commons Attribution 4.0 International License, which permits use, sharing, adaptation, distribution and reproduction in any medium or format, as long as you give appropriate credit to the original author(s) and the source, provide a link to the Creative Commons license, and indicate if changes were made.
In cases where reviewers are anonymous, credit should be given to 'Anonymous Referee' and the source.
The images or other third party material in this Peer Review File are included in the article’s Creative Commons license, unless indicated otherwise in a credit line to the material. If material is not included in the article’s Creative Commons license and your intended use is not permitted by statutory regulation or exceeds the permitted use, you will need to obtain permission directly from the copyright holder.
To view a copy of this license, visit https://creativecommons.org/licenses/by/4.0/
